# A Multi-Sensor Mini-Bioreactor to Preselect Silage Inoculants by Tracking Metabolic Activity *in situ* During Fermentation

**DOI:** 10.3389/fmicb.2021.673795

**Published:** 2021-08-12

**Authors:** Guilin Shan, Victoria Rosner, Andreas Milimonka, Wolfgang Buescher, André Lipski, Christian Maack, Wilfried Berchtold, Ye Wang, David A. Grantz, Yurui Sun

**Affiliations:** ^1^Department of Agricultural Engineering, University of Bonn, Bonn, Germany; ^2^ADDCON GmbH, Bitterfeld-Wolfen, Germany; ^3^Institute of Nutrition and Food Science, University of Bonn, Bonn, Germany; ^4^Department of Botany and Plant Sciences, Kearney Agricultural Center, University of California, Riverside, Riverside, CA, United States

**Keywords:** lactic acid bacteria (LAB), multi-sensor mini-bioreactor (MSMB), fermentation, silage additive, metabolic sensitivity, pH, carbon dioxide (CO_2_), ethanol (EtOH)

## Abstract

The microbiome in silage may vary substantially from the onset to the completion of fermentation. Improved additives and inoculants are being developed to accelerate the ensiling process, to enhance fermentation quality, and to delay spoilage during feed-out. However, current methods for preselecting and characterizing these amendments are time-consuming and costly. Here, we have developed a multi-sensor mini-bioreactor (MSMB) to track microbial fermentation *in situ* and additionally presented a mathematical model for the optimal assessment among candidate inoculants based on the Bolza equation, a fundamental formula in optimal control theory. Three sensors [pH, CO_2_, and ethanol (EtOH)] provided data for assessment, with four additional sensors (O_2_, gas pressure, temperature, and atmospheric pressure) to monitor/control the fermentation environment. This advanced MSMB is demonstrated with an experimental method for evaluating three typical species of lactic acid bacteria (LAB), *Lentilactobacillus buchneri* (LB) alone, and LB mixed with *Lactiplantibacillus plantarum* (LBLP) or with *Enterococcus faecium* (LBEF), all cultured in De Man, Rogosa, and Sharpe (MRS) broth. The fermentation process was monitored *in situ* over 48 h with these candidate microbial strains using the MSMB. The experimental results combine acidification characteristics with production of CO_2_ and EtOH, optimal assessment of the microbes, analysis of the metabolic sensitivity to pH, and partitioning of the contribution of each species to fermentation. These new data demonstrate that the MSMB associated with the novel rapid data-processing method may expedite development of microbial amendments for silage additives.

## Introduction

Silage is a major feedstuff for ruminant animal production worldwide. The biochemical production of silage relies on anaerobic lactic acid fermentation. Homofermentative lactic acid bacteria (LAB) ferment glucose to lactic acid as the primary by-product, whereas heterofermentative LAB ferment glucose to lactic acid, acetic acid, ethanol (EtOH), and carbon dioxide (CO_2_) (Muck, 2004; Kung et al., 2018a; Muck et al., 2018). Testing for heterofermentative fermentation generally involves gas phase sampling of CO_2_ and EtOH (McEniry et al., 2011; Li et al., 2017; Kung et al., 2018b).

Though silage is a nutritious and palatable animal feed, its aerobic deterioration is inevitable when the silo is opened for feeding out, but spoilage can be reduced significantly using either biological or chemical additives (Ranjit and Kung, 2000; Borreani and Tabacco, 2010; Tabacco et al., 2011; Wilkinson and Davies, 2012). Commercial silage inoculants contain highly selected bacteria that speed up silage acidification during anaerobic fermentation (Queiroz et al., 2013; Muck et al., 2018). This typically involves strains of facultative heterofermentative bacteria such as *Lactiplantibacillus plantarum* (LP), and obligate heterofermentative bacteria such as *Lentilactobacillus buchneri* (LB) are used to enhance the aerobic stability of silage (Bolsen et al., 1996; Kung et al., 2018a; Muck et al., 2018). Rapid acidification significantly inhibits the growth of undesirable microorganisms and reduces fermentative loss, while acetic acid, the by-product of heterofermentation, suppresses the aerobic proliferation of spoilage-causing fungi and minimizes oxidative losses during feed-out (Pahlow et al., 2003; Muck, 2004; Kung et al., 2018b).

To continuously improve these biological silage additives requires selection of improved microbial strains from among the abundant species and strains of LAB that are present in natural populations (Muck et al., 2018). Selection is commonly performed during the natural ensiling process, which is time-consuming, laborious, and costly, requiring large numbers of *ex situ* samples and intensive laboratory analyses (Weinberg and Ashbell, 2003). To resolve these bottlenecks in research for silage inoculants, this study presents a multi-sensor-based screening method with three major objectives: (i) to present an alternative model for selecting microbes, (ii) to devise a multi-sensor mini-bioreactor (MSMB) for screening microbial strains during fermentation *in situ*, and (iii) to evaluate the capabilities of the advanced MSMB in selection among candidate inoculants.

## Materials and Methods

### Microbial Selection and Optimal Fermentation

With the use of control system theory (Sargent, 2000), a LAB fermentation process [*X*(*t*)] can be described as a set of state variables [*x*_1_(*t*) – *x*_3_(*t*)] under microbial control *u*_*m*_ such that

(1) $$\begin{array}{c} \dot{X}=\left[\begin{array}{c} {\dot{x}}_{1}\\ {\dot{x}}_{2}\\ {\dot{x}}_{3} \end{array}\right]=F\,\left[\left(x_{1}\,\left(t\right),x_{2}\,\left(t\right),x_{3}\,\left(t\right),u_{\text{m}},t\right)\right]\\ \mathrm{with}\,t_{0}\leq t\leq t_{f} \end{array}$$

where *x*_1_, *x*_2_, and *x*_3_ refer to the accumulated productions of organic acids (lactic and acetic), CO_2_, and EtOH, respectively, with respect to the initial time (*t*_0_) and the final time (*t*_*f*_) of fermentation. As the function of time (*t*), these state variables can be expressed as the outputs of triple integrators such that

(2) $$\begin{array}{c} x_{1}\,\left(t\right)=\frac{1}{\text{pH}\,\left(t\right)}=\int_{t_{0}}^{t}\left[\text{acid}\right]\,\text{dt}\\ x_{2}\,\left(t\right)=\int_{t_{0}}^{t}\left[{\text{CO}}_{2}\right]\,\text{dt}\\ x_{3}\,\left(t\right)=\int_{t_{0}}^{t}\left[\text{EtOH}\right]\,\text{dt} \end{array}\}$$

where [acid] is the instantaneous production of organic acids (primary lactic and acetic), resulting from LAB fermentation. Similar representations apply to carbon dioxide [CO_2_] and [EtOH]. According to optimal control theory (Sargent, 2000), an optimal/minimum cost function of Ĵ subject to Eq. 2 exists, and an optimal control (ȗ_*m–*_*_*best*_*) fulfills

(3) $$\hat{\text{J}}\,\left(\,{ȗ}_{\text{m}-b\,e\,s\,t}\right)=\min.\left(x_{1}+x_{2}+x_{3}\right)=\min.{\left(t_{f}-t_{0}\right)}_{\text{pH}↓}+\min.\int_{t_{0}}^{\min.t_{f}}\left[{\text{CO}}_{2}\,\left(t\right)\right]\,\text{dt}+\min.\int_{t_{0}}^{\min.t_{f}}\left[\text{EtOH}\,\left(t\right)\right]\,\text{dt}$$

where min.(*t*_*f*_ –*t*_0_)_*pH*__↓_ is the shortest time of acidification dynamics of Eq. 1, i.e., the optimal time to be determined by fermentation. The two integrations of *x*_2_(*t*) and *x*_3_(*t*) are related to minimum fermentative loss. Eq. 3 is a special case of the generalized problem of Bolza (Clarke, 1976; Sargent, 2000), which contains a global solution of the time-energy optimization subject to an optimal control function (ȗ_*m–*_*_*best*_*). In this study, the optimal time-energy trajectories of fermentation were determined experimentally by selecting candidate microbes.

### Multi-Sensor-Based Experimental System

**FIGURE 1:**
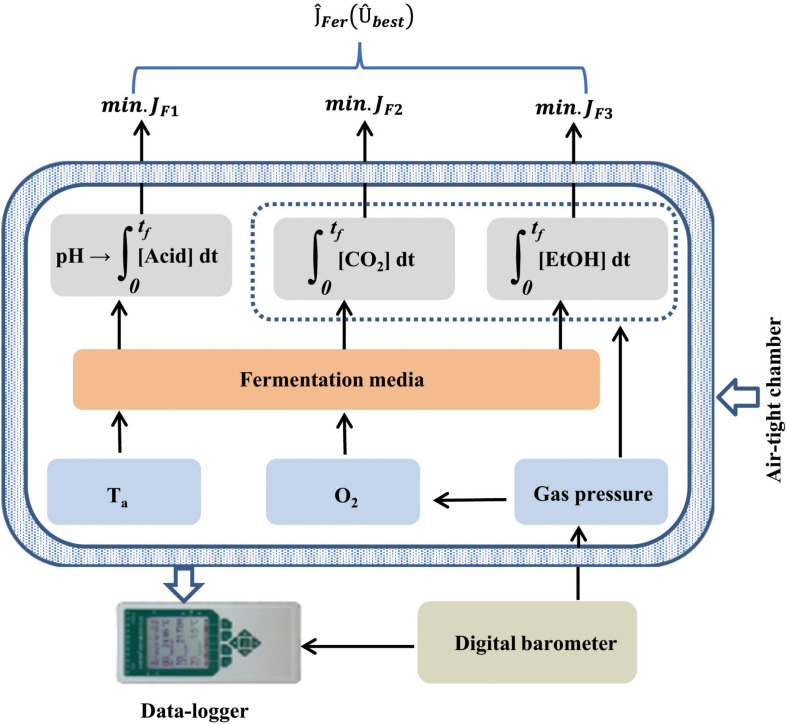
The multi-parameter measurement system devised for selecting microbes used as silage inoculant, based on the characteristics of lactic acid bacterial (LAB) fermentation and the theory of optimal control.

The instrumental structure of the MSMB (Figure 1) contains seven different functional sensors (Table 1), with six enclosed in an air-tight chamber. According to Eq. 2, pH, CO_2_, and EtOH are the three indicators of the LAB fermentation, each corresponding to an analog integrator. Additionally, micro-environmental parameters, i.e., O_2_ concentration, ambient temperature (*T*_*a*_), and gas pressure (*P*_*gas*_) in the sealed chamber, were measured during the fermentation process. *P*_*gas*_ was measured relative to ambient air pressure (*P*_*air*_), which was measured using a digital barometer placed on the outside of the sealed chamber (Figure 1). Throughout the test, *P*_*gas*_ remains positive (*P*_*gas*_ > *P*_*air*_) due to accumulation of CO_2_ and volatile EtOH. The *P*_*gas*_ measurement has two functions: (i) to determine the seal characteristics of the chamber before and during the experiment and (ii) to compensate the O_2_ measurement since the optical O_2_ sensor was calibrated under *P*_*air*_.

**TABLE 1:** Technical information of the pH, CO_2_, ethanol (EtOH), O_2_, temperature, and pressure sensors used.

Parameter	Sensor-type	Manufacturer	Range	Accuracy	Response(s)
pH	BlueLine 21	SI Analytic GmbH, Germany	2–13	±0.3	<20
Carbon dioxide	Prime 2	Clairair Ltd., United Kingdom	0–5% (vol.)	±3% of full scale	<60
Ethanol	TGS2610	Figaro USA, Inc.	500–10,000 (ppm)	±10% of final value in range	<300
Oxygen	OXROB10	Pyro Science GmbH, Germany	Gas phase: 0–100% (vol.)	Gas phase: ± 0.2%	Gas phase: < 7
			Dissolved oxygen:0–44 mg/L	Dissolved oxygen: ± 0.1 mg/L	Dissolved oxygen: < 15
Temperature	FTA15 NiCr-Ni ZA9020FS	Ahlborn Mess-und Regelungstechnik GmbH, Germany	−50 to 200°C	± 0.1°C	1.5
Atmospheric pressure	FDAD12SA	Ahlborn Mess-und Regelungstechnik GmbH, Germany	700–1,050(mbar)	± 0.5% of final value in range	<5
Gas pressure	FDA612SR	Ahlborn Mess-und Regelungstechnik GmbH, Germany	± 1,000(mbar)	±0.5% of final value in range	<5

**FIGURE 2:**
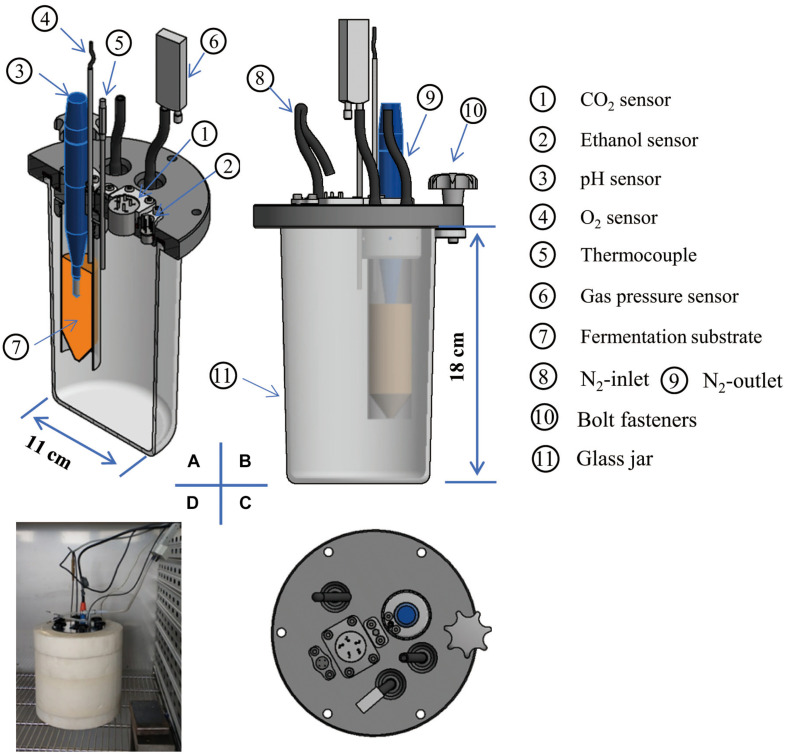
A novel multi-sensor jar, manufactured according to Figure 1, is shown in cross section **(A)**, front view **(B)**, and top view **(C)** and measured *in situ* in the incubator **(D)**.

The MSMB including the air-tight chamber (glass jar, 1.5 L) is schematically shown in Figure 2. A centrifuge tube (inner diameter 2.5 cm, height 12 cm) was filled with fermentation medium (orange color) for culturing the candidate LAB. The pH electrode was immersed in the medium, as the integrator to determine the acidification characteristics of the fermentation relative to each strain or species. The resulting gas from the fermentation diffuses out of the centrifuge tube through six holes (diameter 2 mm) in the wall of the upper tube. Because CO_2_ is unreactive with EtOH, this allows the glass jar to act as a dual integrator for simultaneous collection of the CO_2_ and the volatile EtOH from the fermentation process in the centrifuge tube. The O_2_ sensor can be moved vertically to measure the O_2_ concentration in the gas space or O_2_ dissolved in the medium. To remove O_2_ for anaerobic requirements, paired holes (diameter 3 mm) were perforated in the lid (Figure 2B) for purging with N_2_. Three sets of the MSMB provided replication for simultaneous testing. Three data loggers were linked to (i) the thermocouples and pH electrodes (ALMEMO-2890-9, nine-channel, Ahlborn Mess-und Regelungstechnik GmbH, Ilmenau, Germany), (ii) CO_2_ and EtOH sensors (own manufacture, eight-channel), and (iii) the O_2_ sensor (four-channel, Pyro Science GmbH, Aachen, Germany), all sampled at 10-min intervals.

### Sample Preparation

Strains of typical species of obligate heterofermentative bacteria, *Lt. buchneri* DSM 13573 (LB) and two typical species of facultative heterofermentative bacteria, *Lp. plantarum* DSM 3676, *Lp. plantarum* DSM 3677, and *Enterococcus faecium* NCIMB 11181 (EF), were chosen for the experiment. The two strains of *Lb. plantarum* were used as a mixture (1:1) in all experiments and designated as LP. *Lt. buchneri* DSM 13573 (LB) was used individually and mixed with *Lp. plantarum* DSM 3676/DSM 3677 (LBLP) or with *E. faecium* NCIMB 11181 (LBEF).

All candidate bacteria were prepared as lyophilizates by cultivating the strains on synthetic medium and harvesting the cells by centrifugation. The harvested biomass was lyophilized at −40°C for 2 days; and the lyophilizates were stored at −18°C. The cell density of the lyophilized LB was 1.6 × 10^12^ CFU/g. As 0.1 g of the lyophilized LB with 40 ml of De Man, Rogosa, and Sharpe (MRS) (Table 2) was cultured during the experiment, the resulting inoculum density of LB was 4 × 10^9^ CFU/ml. For LBLP and LBEF, either *Lp. plantarum* DSM 3676/DSM 3677 or *E. faecium* NCIMB 11181 was added with 10% of the cell density of the LB (i.e., 4 × 10^8^ CFU/ml). The cell density of the lyophilized LP was 1 × 10^11^ CFU/g, and that of the lyophilized EF was 3 × 10^12^ CFU/g. Thus, 0.160 g of the lyophilized LP and 0.005 g of the lyophilized EF were mixed with 0.1 g of the lyophilized LB. These microbial samples, in 40 ml of sterile MRS broth in each centrifuge tube (vol. 50 ml), were incubated inside the multi-sensor instrumented jar at 30°C for 48 h.

**TABLE 2:** Composition of the De Man, Rogosa, and Sharpe (MRS) broth.

Substance	Con.	Substance	Con.	Substance	Con.
Peptone	10 g/l	Yeast extract	4 g/l	Dipotassium phosphate	2 g/l
Glucose	20 g/l	Sodium acetate	5 g/l	Ammonium citrate	2 g/l
Beef extract	8 g/l	Polysorbate 80	1 g/l	Magnesium sulfate (MgSO_4_)	0.2 g/l

*MRS, De Man, Rogosa, and Sharpe.*

### Chemical Analyses

All the fermented samples were frozen in the centrifuge tubes at −20°C immediately after incubation, prior to chemical analyses. The fermentation acids (lactic and acetic), EtOH, and propanediol were determined using high-performance liquid chromatography (HPLC; KNAUER Azura, Wissenschaftliche Geräte GmbH, Berlin, Germany), coupled with integrated UV and refractive index (RI) detectors as described by Shan et al. (2019).

### Signal Processing

#### Normalized Productions of $\bar{\text{C}\,O_{2}}$ and $\bar{\text{E}\,\mathrm{tOH}}$

To compare the relative rate of increase of the CO_2_ or EtOH in the fermentation process, the normalized productions of carbon dioxide ($\bar{{\text{CO}}_{2}}$) and $\bar{\text{EtOH}}$ were calculated, respectively, as

(4) $$\bar{{\text{CO}}_{2}\,\left(t\right)}=\frac{\int_{0}^{t}\left[{\text{CO}}_{2}\right]\,dt}{\int_{0}^{t_{f}}\left[{\text{CO}}_{2}\right]\,dt}\;0\leq t\leq t_{f}$$

and

(5) $$\bar{\text{EtOH}\,\left(t\right)}=\frac{\int_{0}^{t}\left[\text{EtOH}\right]\,dt}{\int_{0}^{t_{f}}\left[\text{EtOH}\right]\,dt}\;0\leq t\leq t_{f}$$

where both $\bar{{\text{CO}}_{2}}$ and $\bar{\text{EtOH}}$ vary from 0 to 1 as functions of *t*.

#### Temporal Rate of ΔCO_2_ and ΔEtOH

This differential variable may reflect the dynamics of metabolic activity of microorganisms. As sequences of discrete-time data, the differentials of CO_2_ and EtOH were calculated over time as

(6) $$\begin{array}{c} △\,{\text{CO}}_{2}=\left[{\text{CO}}_{2}\,\left(t_{i}\right)\right]-\left[{\text{CO}}_{2}\,\left(t_{i-1}\right)\right]\\ △\,\text{EtOH}=\left[\text{EtOH}\,\left(t_{i}\right)\right]-\left[\text{EtOH}\,\left(t_{i-1}\right)\right]\\ t_{0}=0,t_{n}=t_{f},i=1,2,\ldots .n \end{array}\}$$

#### Signal Decomposition

Signal decomposition, a function of smart instruments, is often used to partition a mixed source signal into its constitutive pure components for various engineering problems (Li et al., 2013; Shan et al., 2019). In this study, the fermentation characteristics relative to different strains or combinations are regarded as mathematical curves in functional space. Three time courses of pH (i.e., pH_*LB*_, pH_*LBLP*_, and pH_*LBEF*_) are directly tracked *in situ* from the experiment. Through data decomposition, pH_*LP*_ and pH_*EF*_ can also be obtained, such that

(7) $$\begin{array}{c} {\text{pH}}_{\text{LP}}\,\left(t\right)={\text{pH}}_{0}+\left({\text{pH}}_{\text{LBLP}}\,\left(t\right)-p\,{\text{H}}_{\text{LB}}\,\left(t\right)\right)\\ {\text{pH}}_{\text{EF}}\,\left(t\right)={\text{pH}}_{0}+\left({\text{pH}}_{\text{LBEF}}\,\left(t\right)-p\,{\text{H}}_{\text{LB}}\,\left(t\right)\right) \end{array}\}$$

where pH_0_ is the initial value of each substrate.

### Statistical Analysis

The experimental data were analyzed using IBM SPSS v25.0 (IBM Co., Armonk, NY, United States). Linear regression, curve fitting, and fitting errors were evaluated using coefficient of determination (*R*^2^) and root mean square error (RMSE). Two-way analysis of variance (ANOVA) was conducted for effects of the experimental scheme (two air environments, i.e., anaerobic and aerobic), treatment (three types, i.e., LB, LBEF, and LBLP), and the interactions of the chemical compositions for the final-data processing. One-way ANOVA was used to evaluate the statistical significance among anaerobic and aerobic environments.

## Results

### Acidification Characteristics

**FIGURE 3:**
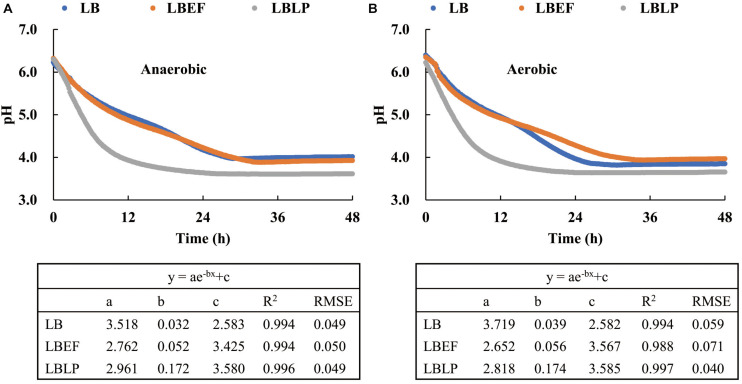
Three time courses of the acidification process with respect to *Lentilactobacillus buchneri* (LB), LB mixed with *Enterococcus faecium* (LBEF), and LB mixed with *Lactiplantibacillus plantarum* (LBLP), under anaerobic **(A)** and aerobic conditions **(B)**. LB, *Lentilactobacillus buchneri*; LBEF, LB mixed with *Enterococcus faecium*; LBLP, LB mixed with *Lactiplantibacillus plantarum*.

**FIGURE 4:**
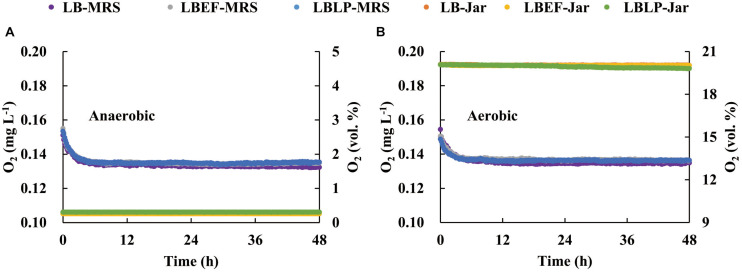
Time courses of O_2_ concentration dissolved in the De Man, Rogosa, and Sharpe (MRS) and distributed in the sealed jars during the anaerobic **(A)** and aerobic **(B)** fermentations. MRS, De Man, Rogosa, and Sharpe.

Two sets of time courses of pH (Figure 3), each with respect to the mean of three replicates, were recorded *in situ* from the fermentation process in the MSMB, the first set (Figure 3A) from anaerobic fermentation and the second set (Figure 3B) from aerobic fermentation. The patterns observed were similar. Figure 4 shows the time courses of O_2_ concentration over the experiment with two parts, i.e., as liquid phase dissolved in the MRS and as gaseous phase distributed in the glass jars. When comparing the anaerobic fermentation (Figure 4A) and aerobic one (Figure 4B), the amounts of gaseous oxygen in these jars remained two constant levels throughout the experiment, i.e., around 0.2 vol. % of O_2_ concentration in the anaerobic jars and 20 vol. % of O_2_ concentration in the aerobic jars. The O_2_ dissolved in the MRS in both anaerobic and aerobic jars varied with similar trends in the initial 5 h, i.e., declined from 0.157 to 0.138 mg/L, and then reached a plateau. This and the chemical analyses from aerobic and anaerobic fermentations at the end of the experiment (Table 3) demonstrate that the oxygen in the MSMB had minimal impact on the respiratory metabolism of the microbes tested.

**TABLE 3:** Final chemical analyses of the three types of samples.

Variable	Anaerobic			Aerobic				Significance of effects		
	LB	LBEF	LBLP	LB	LBEF	LBLP	SEM	A	T	A × T
pH	4.06^a^	4.07^a^	3.61^b^	4.10^A^	3.96^A^	3.65^B^	0.048	NS	**	NS
Lactic acid (g/l)	13.56	12.23	16.44	12.77	13.52	15.33	1.238	NS	NS	NS
Acetic acid (g/l)	5.74	5.27	4.82	5.78	6.08	4.66	0.420	NS	NS	NS
Ethanol (g/l)	3.36^a^	1.81^b^	1.08^c^	3.47^A^	1.95^B^	1.00^C^	0.133	NS	**	NS
Propanediol (g/l)	0.327^a^	0.317^a^	0.253^b^	0.397^A^	0.453^A^	0.207^B^	0.037	NS	**	NS

SEM, standard error of the mean; A, air environment (anaerobic or aerobic); T, treatment (LB, LBEF, or LBLP). LB, *Lentilactobacillus buchneri*; LBEF, LB mixed with *Enterococcus faecium*; LBLP, LB mixed with *Lactiplantibacillus plantarum*; NS, not significant. **p < 0.01. ^*a*–*c*,^^*A*–*C*^Means with different superscripts within the same air environment differ significantly (*p* < 0.05).

In general, all time courses of pH (Figures 3A,B), i.e., the acidification characteristics, were well described by exponential regressions, in both anaerobic and aerobic fermentations. The steepest decline was observed from the LBLP (pH_*LBLP*_ = 5, *t* = 4.5 h, anaerobic), evidently faster than that of LBEF (pH_*LBEF*_ = 5, *t* = 10 h, anaerobic) or that of the LB (pH_*LB*_ = 5, *t* = 11.5 h, anaerobic). Additionally, the pH_*LBLP*_ had the lowest values (pH_*LBLP*_ = 3.61 or 3.65) in both anaerobic and aerobic fermentations, which agreed with the finding that the contents of lactic acid in the fermented MRS of LBLP are slightly higher than those of LB and LBEF (Table 3).

The final time (*t*_*f*_) was determined as pH ≤ 4 over six consecutive measurements (i.e., 1 h). The *t*_*f*_ of the pH_*LBLP*_ was only 12 h, whereas the *t*_*f*_ for both pH_*LBEF*_ and pH_*LB*_ was more than double at 28 h. Therefore, for these microbial samples in the same experimental conditions, the acidification characteristics of the LBLP had the lowest *t*_*f*_ (i.e., the fastest process). Therefore, the resulting time course of pH_*LBLP*_ is time optimal, but this only fulfilled by one of the two necessary criteria for a global optimal solution of the Bolza equation (i.e., the term of min.(*t*_*f*_ – *t*_0_)**_*pH*__↓_** in Eq. 3).

### Formations of CO_2_ and EtOH

**FIGURE 5:**
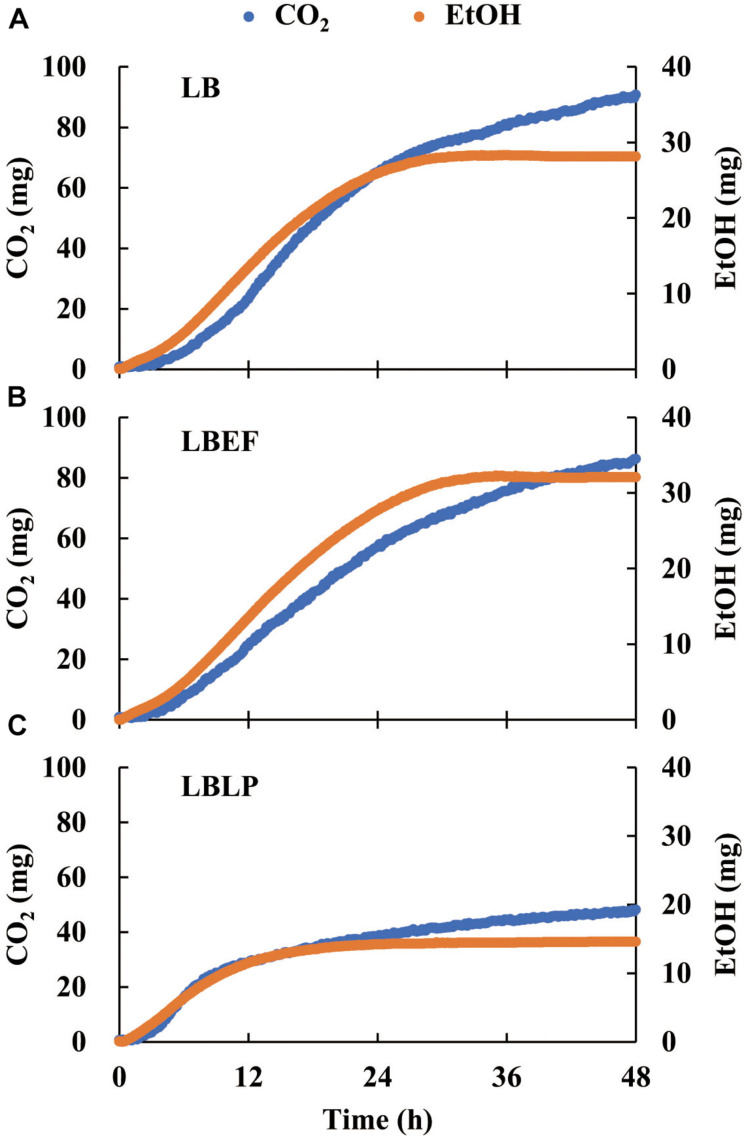
Time courses of CO_2_ and ethanol production with respect to LB **(A)**, LBEF **(B)** and LBLP **(C)** under anaerobic conditions. LB, *Lentilactobacillus buchneri*; LBEF, LB mixed with *Enterococcus faecium*; LBLP, LB mixed with *Lactiplantibacillus plantarum*.

**FIGURE 6:**
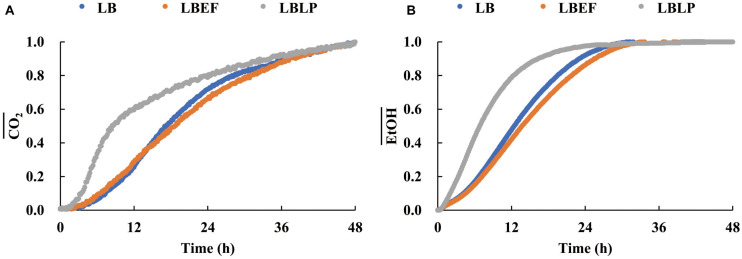
Relative variations of CO_2_ **(A)** and EtOH **(B)** productions of the three samples.

Three time courses of CO_2_ and EtOH formation (Figure 5) were recorded from the LB (A), the LBEF (B), and the LBLP (C). The fermentation governed by the LBLP yielded the lowest CO_2_ (48.12 mg) and EtOH (14.55 mg) in the period of 48 h. In contrast, the fermentation with the LB or the LBEF yielded more than twice as much CO_2_ (86.21–90.72 mg) and EtOH (28.14–32.10 mg). Figure 6 shows the normalized CO_2_ production ($\bar{{\text{CO}}_{2}}$, Figure 6A) and $\bar{\text{EtOH}}$ (Figure 6B) from the three samples. For the LBLP, the $\bar{{\text{CO}}_{2}}$ reached 0.8 of the final production in 24.5 h, while the $\bar{{\text{CO}}_{2}}$ = 0.8 for the LB was 28 h or for the LBEF was 31 h. Similarly, the transition times of $\bar{\text{EtOH}}$ increased to 0.8 for the LBLP, LB, and LBEF at 12.3, 19.5, and 21.8 h, respectively. In contrast, the rate of EtOH increase was greater than that of CO_2_ in all the samples.

### Metabolic Sensitivity to pH

**FIGURE 7:**
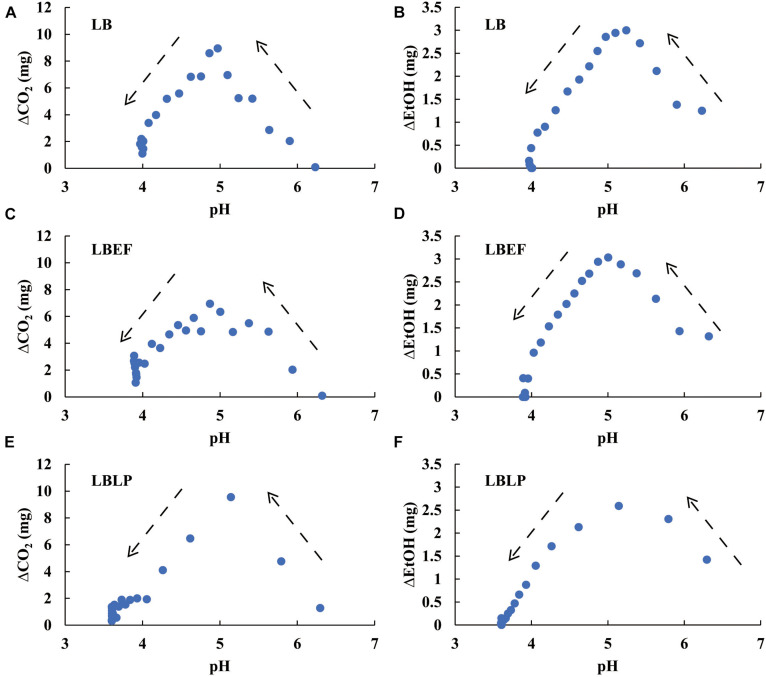
The relationships between the pH of the three microbial samples and the differential variables of both CO_2_ production **(A,C,E)** and EtOH production **(B,D,F)**, where ΔCO_2_ = CO_2_ (*t*_*i*_) − CO_2_ (*t*_*i*__–__1_) and ΔEtOH = EtOH(*t*_*i*_) − EtOH(*t*_*i*__–__1_), both calculated with *t*_*i*_ − *t*_*i*__–__1_ = 2 h, *i* = 0, 1, …,*n*. The arrows denote the time course of decreasing pH.

**FIGURE 8:**
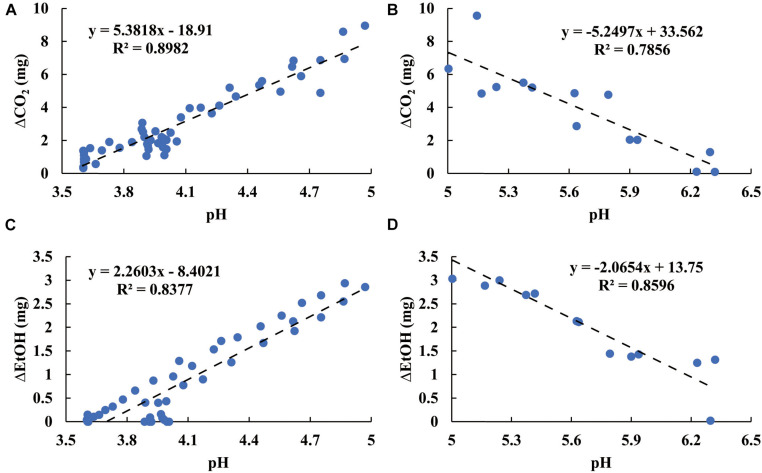
Effect of pH on the rate of microbial respiration in relation to ΔCO_2_ with pH < 5 **(A)**, ΔCO_2_ with pH ≥ 5 **(B)**, ΔEtOH with pH < 5 **(C)**, and ΔEtOH with pH ≥ 5 **(D)**.

Figure 7 shows stepwise tracing (*t*_*i*_ − *t*_*i*__–__1_ = 2 h, Eq. 6) for the temporal production of both CO_2_ and EtOH. In the initial period of fermentation (pH > 5), the increasing ΔCO_2_ and ΔEtOH reflected the increasing microbial activity for all microbes. A turning point of ΔCO_2_ and ΔEtOH occurred around pH 5. Below pH 5, the metabolic activity decreased as the pH declined. After the pH declined to 4, both ΔCO_2_ and ΔEtOH reached minima and then achieved steady state. The patterns in Figure 7 not only characterize the metabolic sensitivity of these microbes to pH but also contain the dynamic information of the acidification process from each sample. For the LBLP sample, pH_*LBLP*_ decreased to 5 in less time (4.5 h), resulting in only three data points of ΔCO_2_ (*t*_*i*_ - *t*_*i*__–__1_ = 2 h) while pH ≥ 5. In contrast, seven data points of ΔCO_2_ for LB and LBEF were recorded due to the longer periods of pH ≥ 5 (pH_*LB*_, 11.5 h; pH_*LBEF*,_ 10 h). The patterns of EtOH (Figures 7B,D) had similar temporal implications. Because the turning points of ΔCO_2_ and ΔEtOH at pH 5 correlated for all the microbial samples (Figure 7), the general effect of pH on the metabolic activity can be estimated. Figure 8 presents four piecewise linear regressions corresponding to ΔCO_2_ (Figure 8A pH < 5, Figure 8B pH > 5) and ΔEtOH (Figure 8C pH < 5, Figure 8D pH > 5), related to the three microbial samples.

### Role Partition of Each Strain

**FIGURE 9:**
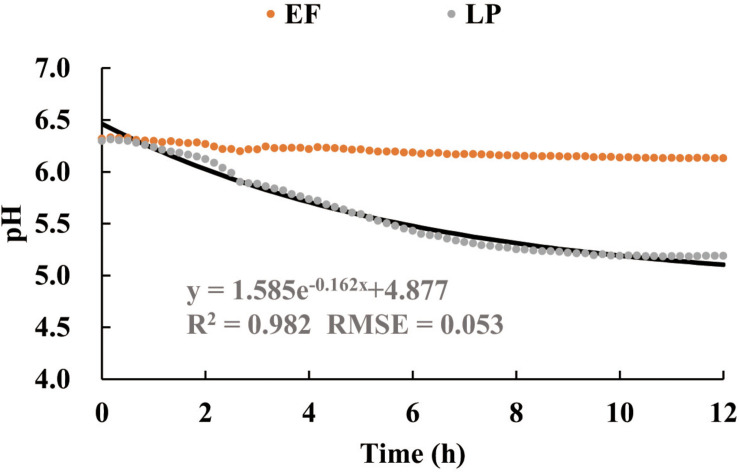
The data decomposition of the time courses of pH in Figure 3A to separate the additional roles of *Enterococcus faecium* (EF) or *Lactiplantibacillus plantarum* (LP) to support LB in accelerating the acidification process of the substrate (MRS). The solid line is the regression approximation to the separate data of LP. EF, *Enterococcus faecium*; LP, *Lactiplantibacillus plantarum*; LB, *Lentilactobacillus buchneri*; MRS, De Man, Rogosa, and Sharpe.

In functional space, the time courses of both pH_*LBLP*_ and pH_*LBEF*_ (Figure 3) are mathematically decomposable in relation to the time course of pH_*LB*_. With the use of Eq. 7 together with the time course of pH_*LB*_ (Figure 3) as reference, Figure 9 shows these separate time courses for pH_*LP*_ and pH_*EF*_ over the initial 12 h of the fermentation. We presented the decomposed data in the early stage (0–12 h) because pH_*LBLP*_ reached a plateau within 12 h ≤ *t* ≤ 48 h (Figure 3). The regression demonstrated that the LP species played an exponentially accelerating role (R^2^ = 0.982, RMSE = 0.053) in the acidification process over pH ranging from 6.23 to 5.0. The major contribution of LP to the fermentation process was observed in the initial period of 0–6 h, i.e., pH ≥ 5.5. Subsequently, the accelerating role of the LP attenuated quickly as the pH decreased, and this could be attributed to the stronger suppression from the increasing organic acid in the fermentation medium. Alternatively, the separate role of the EF is a straight line, perpendicular to the pH axis at the initial point (pH_0_ = 6.23). This demonstrates that the role of the EF in accelerating the acidification process is negligible, not an optimal solution for Eq. 3.

## Discussion

Both the shortest acidification process (min. *t*_*f*_ in section “Acidification Characteristics”) and the minimum gas production (see section “Formations of CO_2_ and EtOH”) resulted from the LBLP, and therefore, this is a time-energy optimal fermentation reaction (Sargent, 2000). Moreover, the time course of pH_*LBLP*_ (Figure 3) is the optimal trajectory of the fermentation process, and the exponential equation of the LP (Figure 9) is the optimal control function (ȗ_*m–*_*_*best*_*) determined. The paired optimal solutions were found experimentally and subject to the Bolza equation (Eq. 3) (Clarke, 1976).

As expected, lactic acid was the primary metabolite of LAB fermentation (Table 3). Our experimental data supported the hypothesis that pH was primarily dominated by variations in lactic acid during the LAB fermentation process (Kung et al., 2018a) from two notes: (i) the *Lp. plantarum* strains DSM 3676 and DSM 3677 (LP), the facultative heterofermentative strains, played a major role in producing lactic acid during the initial 0–6 h of the fermentation (Figures 3, 9). At *t* = 6 h, the pH_*LBLP*_ = 4.62 indicated that most lactic acid had already been formed. (ii) The pKa of acetic acid (4.75) is higher than that of lactic acid (3.86), reflecting it being a 10 times weaker acid than lactic acid (Danner et al., 2003; Graves et al., 2006; Kung et al., 2018a).

Our data of the relationship between CO_2_ and pH (Figures 7, 8) support a value of pH 5 as a critical value governing microbial growth, with rapid (pH > 5) or slower to no growth (pH < 5) during the ensiling process (Kung et al., 2003; Pahlow et al., 2003). We show this for the first time using the dynamics of microbial respiration over the course of fermentation. This had previously been suggested from an *ex situ* determination of microbial counts (Oliveira et al., 2017). Our *in situ* method obviates the process of plate-culture counts, which create assessment delays of several days (Wilkinson and Muck, 2019) and may require multiple samplings over time. In contrast to the *ex situ* method, our MSMB provides both real-time anaerobic measurements of CO_2_ and EtOH productions and instantaneous microbial activity.

A few studies tested silage inoculants using MRS broth or the aqueous extract of silage crop (Oude Elferink et al., 2001; Danner et al., 2003; Holzer et al., 2003; Graves et al., 2006; Arasu et al., 2015; Blajman et al., 2020). Fermentation characteristics, such as production of organic acids, decreasing dynamics of pH, and CO_2_ production, depend on medium composition (Danner et al., 2003; Blajman et al., 2020). The next step of our study is to replace MRS broth with the aqueous extract of silage, creating a testing condition that may be closer to the natural culture of silage ensiling for LAB. However, the effect of the buffering capacity of silage crop on pH is inherent (Kung et al., 2003; Pahlow et al., 2003; Shan et al., 2021) and should be taken into account. Unfortunately, this effect cannot be evaluated when either MRS broth or the liquid extraction of silage crop is used as fermentation medium.

High concentrations of EtOH are usually attributed to large numbers of yeasts (Kung and Ranjit, 2001; Kung et al., 2018a). However, the silage containing LB (Oude Elferink et al., 2001) produced EtOH from anaerobic degradation of lactic acid in corn silage. Kung and Ranjit (2001) noted the high EtOH from treated barley silage, but not from the control. The EtOH data of our study from the MRS broth support the previous observations. Moreover, in this study, EtOH was measured *in situ* as the gaseous concentration in the sealed jar, which by Henry’s law is directly proportional to the concentration of EtOH dissolved in the fermentation medium (Sun et al., 2015). While the partition coefficient of Henry’s law is temperature-dependent, the fermentation here was carried out at constant temperature (30°C).

During the ensiling process, the CO_2_ recorded in the fermentation phase consists of two parts, one from the initial aerobic phase and one from anaerobic fermentation (Li et al., 2017). It is challenging to separate them into two CO_2_ pools for the different phases (Shan et al., 2019). In this study, the measured CO_2_ (Figures 5–8) derived completely from anaerobic heterofermentative LAB fermentation because the MSMB provided a manageable environment between anaerobic and aerobic seals. This is also an advantage of the MSMB over the ensiling experiment with the natural culture of silage.

It is not surprising that Figures 3A,B had similar patterns. Members of the family Lactobacillaceae are anaerobic, but the majority of species are oxygen tolerant to some degree and often completely. Only very few species of LAB were observed to react to O_2_ (Condon, 1987). On the technical side, Figure 3 demonstrates that the multi-sensor instrument presented here is suitable for both anaerobic and aerobic experiments. Since the aerobic stability of silage refers to a de-acidification process governed by fungus in silage (Wilkinson and Davies, 2012; Kung et al., 2018a), and the aerobic deterioration of silage is commonly associated with changes in temperature, pH, CO_2_ production, and O_2_ consumption (Honig, 1990; Muck and Pitt, 1994; Weinberg and Ashbell, 2003; Sun et al., 2015; Robinson and Swanepoel, 2016), this novel MSMB could also be useful to qualitatively observe the role of acetic acid in inhibiting fungal growth during aerobic phases of silage production.

## Conclusion

We have devised an MSMB to facilitate preselection of microbes as silage inoculants. We demonstrate successful screening of the dynamics of the acidification process, gas production, and metabolic activity from the MRS-based LAB fermentation, using *in situ* simultaneous measurements of pH, CO_2_, and EtOH. We have further used this novel information to introduce an optimal control model, using signal decomposition, for selecting candidate microbes. Future studies are planned to evaluate this novel prototype with aqueous extracts of common silage materials during anaerobic LAB fermentation and to extend its applicability to silage aerobic stability.

## Data Availability Statement

The original contributions presented in the study are included in the article/supplementary material, further inquiries can be directed to the corresponding author.

## Author Contributions

AM and YS designed the study. WBu, GS, WBe, CM, and YS devised the instrument. GS, VR, YW, and YS performed the experiment. YS designed the mathematical frame. GS and YS designed the data process. GS made the statistical analysis. VR conducted the chemical analysis. GS, AM, AL, DG, and YS wrote the manuscript. All authors contributed to the article and approved the submitted version.

## Conflict of Interest

VR and AM were employed by company ADDCON GmbH. The remaining authors declare that the research was conducted in the absence of any commercial or financial relationships that could be construed as a potential conflict of interest.

## Publisher’s Note

All claims expressed in this article are solely those of the authors and do not necessarily represent those of their affiliated organizations, or those of the publisher, the editors and the reviewers. Any product that may be evaluated in this article, or claim that may be made by its manufacturer, is not guaranteed or endorsed by the publisher.
